# Clinical application of robotic telesurgery in the Brazilian public health system: a consecutive case series

**DOI:** 10.1016/j.clinsp.2026.101020

**Published:** 2026-06-09

**Authors:** Everson L.A. Artifon, Felipe Kfouri, Jose Pinhata Otoch, Luiz Paulo Kowalski, William C. Nahas, Paulo Pego-Fernandes, Edmund C. Baracat, Marcos Samano, Ricardo Terra, Sergio C. Ribeiro, Jose Maria Soares, Gustavo Ebaid, Gabriel dos Anjos, Eduardo V. Motta, Pedro Nabuco Xavier, Marco A. Kulcsar, Herbert D. Souza, Alessandro R. Belon, Giovanni G. Cerri

**Affiliations:** a Departamento de Cirurgia, Faculdade de Medicina da Universidade de São Paulo (FMUSP), São Paulo, SP, Brazil; b Departamento de Cardiopneumologia, Faculdade de Medicina da Universidade de São Paulo (FMUSP), São Paulo, SP, Brazil; c Departamento de Ginecologia e Obstetrícia, Faculdade de Medicina da Universidade de São Paulo (FMUSP), São Paulo, SP, Brazil; d Faculdade de Medicina, Universidade de São Paulo (FMUSP), São Paulo, SP, Brazil; e Departamento de Radiologia e Oncologia, INOVA-HC ‒ Núcleo de Inovação Tecnológica do Hospital das Clínicas da Faculdade de Medicina da Universidade de São Paulo (FMUSP), São Paulo, SP, Brazil

**Keywords:** Robotic surgical procedures, Telemedicine, Brazil, National health programs, Patient safety, Feasibility studies

## Abstract

**Objective:**

To report the first clinical application of robotic telesurgery within the Brazilian Unified Health System (SUS) infrastructure, evaluating feasibility, safety, and stakeholder perceptions in a consecutive case series.

**Methods:**

Five consecutive robotic telesurgeries across four surgical specialties were performed using the Toumai/MicroPort platform. The remote surgeon operated from the console at PROMIN-FMUSP, while the robotic unit and patient were located at HU-USP, approximately 5 km away. All procedures used a wired network connection. A structured safety protocol with predefined conversion triggers and a standby bedside surgeon was applied. Outcomes included procedural completion, network latency, intraoperative events, and 30-day postoperative outcomes. Post-procedure structured interviews captured perceptions from the remote surgeon, bedside surgeon, and anesthesia team using Likert scales and the NASA-TLX adapted instrument.

**Results:**

Four of five procedures were completed entirely via remote console. One case (radical prostatectomy) required conversion to local surgeon control due to a local cable disconnection at the patient site near the end of the procedure (the network connection itself was preserved); the bedside surgeon completed the remaining approximately 10 min without adverse consequences. Surgical specialties included thoracic surgery (two cases), urology, head and neck surgery, and gynecology. Median operative time was 165 min (range 106‒240). Console-reported latency was approximately 12 ms in each case. No intraoperative adverse events occurred. All remote surgeons rated perceived safety at the maximum score. Bedside surgeons and anesthesiologists uniformly considered the model safe, although saturation of most perception scores at the maximum value limited inter-case discrimination. One patient (Case 2, head and neck) had a Clavien-Dindo grade II complication (edema and pain requiring one additional day of hospitalization). No readmissions, reoperations, or mortality occurred within 30 days.

**Conclusion:**

The first clinical application of robotic telesurgery within SUS proved technically feasible across four surgical specialties in five consecutive low-to-medium complexity cases, with no major adverse events at 30-days. The single conversion was managed safely by the standby bedside surgeon, consistent with the intended behavior of the structured safety protocol. These initial findings ‒ limited by the small sample and absence of a control group ‒ support the continued, progressive evaluation of telesurgery within Brazil's public health system.

## Highlights

• First clinical application of robotic telesurgery within Brazil's SUS (n = 5).
• Four of five procedures completed entirely via remote console; one safely converted.
• Mean console-extracted latency 12 ms over institutional wired network.
• Favorable perceptions across remote, bedside and anesthesia teams.
• One Clavien-Dindo II complication; no readmission, reoperation or 30-day mortality.

## Introduction

Since the seminal Lindbergh transatlantic operation in 2001,^1^ robotic telesurgery has progressed from experimental demonstrations to early clinical implementation, with recent prospective studies reporting successful remote cholecystectomies, gastrectomies, and hepatectomies using 5G-enabled platforms^2^, ^3^, ^4^^,^^5^ The broader telesurgery field has been recently summarized in a scoping review by Misra et al.^6^ These advances establish the technical feasibility of remote robotic surgery; however, the vast majority of clinical telesurgery data originate from controlled trials in high-resource settings or dedicated 5 G network deployments.^5^^,^^7^^,^^8^ Whether telesurgery can be safely implemented within the existing infrastructure of public health systems ‒ where it could most significantly expand surgical access ‒ remains largely unexplored.

We previously conducted a dry lab feasibility study evaluating the same robotic telesurgery platform (Toumai/MicroPort) over the Brazilian Unified Health System (SUS) network infrastructure, connecting two academic hospitals in São Paulo (PROMIN-FMUSP and HU-USP)^9^ That study demonstrated high task completion rates, favorable surgeon perceptions, and acceptable connectivity metrics under both wired and private 5 G conditions across 28 participants. Based on these findings, and following the development of a structured safety protocol with predefined conversion criteria, we proceeded to the first clinical application of the platform.

The aim of this study was to report the first consecutive case series of robotic telesurgeries performed within SUS infrastructure, evaluating procedural feasibility, network performance, intraoperative safety, postoperative outcomes, and the perceptions of all involved stakeholders ‒ the remote surgeon, the bedside surgical team, and the anesthesia team.

## Patients and methods

### Study design and ethical approval

This was a prospective, dual-site case series of five consecutive robotic telesurgeries conducted between February 24, 2026 and March 3, 2026. The remote console was located at PROMIN-FMUSP and the robotic unit and patient at HU-USP. The study was approved by the institutional ethics committee (CAAE 94,555,325.3.0000.0076), conducted in accordance with the Declaration of Helsinki, and is reported following the PROCESS 2025 guideline for case series^10^

### Patient and team consent

Written informed consent for the telesurgical approach and for the use of anonymized data for research purposes was obtained from each patient using a study-specific consent form, approved by the institutional ethics committee and distinct from the standard surgical consent. The form disclosed the experimental nature of the procedure, the geographic separation between the patient and the remote operator, the predefined network-failure scenarios that could trigger conversion to the bedside surgeon, and the patient's right to refuse the telesurgical approach without prejudice to receiving conventional robotic or open surgery. The identity and individual qualifications of the operating team members were communicated verbally to the patient during the consent encounter and recorded in the medical chart, but were not transcribed into the written form, in alignment with institutional consent practice; this approach was reviewed and approved by the institutional ethics committee. Consent was obtained by a study investigator on the day before the procedure. The consent process was designed in alignment with the ethical framework proposed by Frenkel et al. for elective robotic telesurgery.^11^ All participating surgeons, bedside teams, and anesthesiologists provided informed consent for the structured post-procedure interviews.

### Setting and telesurgery platform

**Fig. 1:**
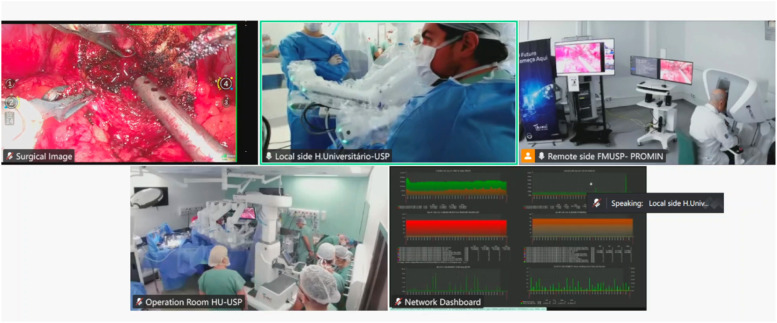
Integrated real-time monitoring display at the remote site (PROMIN-FMUSP) during a clinical telesurgery. The unified screen combined live video from cameras within the remote console room (FMUSP), live video from cameras in the operating room at Hospital Universitário da Universidade de São Paulo (HU-USP) showing the robotic unit and bedside team, the live endoscopic view from the robot displaying the surgical field, and a connectivity panel reporting network status. This consolidated layout supported continuous situational awareness across both sites, direct visualization of the ongoing procedure, and uninterrupted monitoring of network performance throughout each procedure.

The telesurgery setup was identical to our previously described dry lab configuration^9^ The remote surgeon operated the Toumai® robotic surgical system console (MicroPort® MedBot, Shanghai, China) installed at PROMIN-FMUSP, while the robotic unit and patient were located in the operating room at Hospital Universitário da Universidade de São Paulo (HU-USP), approximately 5 km away (Fig. 1). All procedures were performed using the institutional wired network connection. The private 5 G network was not used in the clinical phase.

### Patient selection

Patients were selected based on the following criteria:

− Elective surgical indication amenable to robotic approach.
− ASA physical status I‒III.
− Case complexity classified as low to medium.
− Informed consent for telesurgical approach.
− Availability of a qualified bedside surgeon capable of completing the procedure independently in case of conversion.

### Safety protocol

A structured safety protocol was developed based on international telesurgery guidelines and our dry lab experience.^9^^,^^12^ Conversion criteria were defined a priori but operationalized through the remote surgeon's real-time judgment rather than fixed numerical thresholds. They included: subjectively unacceptable latency or video/audio degradation perceived to compromise safe surgical control, unrecoverable loss of video or command, or any clinical indication requiring immediate local intervention. In the event of a conversion trigger, the bedside surgeon assumed immediate control of the procedure. Conversion was communicated verbally between sites, and the remote surgeon remained available at the console to resume the procedure if the technical issue was resolved.

Although activation of conversion was triggered by the remote surgeon's clinical judgment rather than by fixed numerical thresholds ‒ a design choice consistent with conversion decisions in conventional robotic surgery and aligned with the framework recommended by Wang et al.^12^ ‒ the categories of trigger (technical vs. clinical), the conversion workflow, the verbal communication protocol, and the standby-bedside-surgeon role were defined and rehearsed prospectively prior to the first clinical case.

### Surgical team composition

Each procedure involved:

− A remote surgeon at the console (PROMIN-FMUSP), experienced in robotic surgery and trained on the Toumai platform.
− A bedside surgeon at HU-USP, experienced in robotic surgery and capable of independently completing the procedure.
− A dedicated anesthesia team at HU-USP.
− A clinical engineering/IT support team monitoring network performance in real time at both sites.

### Network performance monitoring

Network connectivity was monitored continuously using the robotic system's built-in indicators. A single representative latency value was read from the console's connectivity display by the remote surgeon at the end of each procedure, at the resolution displayed by the console; continuous instrumented logging of jitter and packet loss, performed during Phase 1, was not maintained during clinical procedures and is acknowledged as a methodological limitation. The occurrence of video freezes, audio dropouts, and loss of command was logged prospectively.

### Outcome measures

Primary outcomes:

− Successful completion of the procedure via remote console (no conversion).
− 30-day postoperative complications (Clavien-Dindo classification)^13^

Secondary outcomes:

− Operative and console times.
− Intraoperative network metrics (representative latency).
− Intraoperative adverse events and safety trigger activations.
− Perceptions of the remote surgeon (NASA-TLX adapted workload, system perception, safety, clinical acceptability).
− Perceptions of the bedside surgeon (readiness, communication, patient safety, confidence).
− Perceptions of the anesthesia team (hemodynamic stability, patient safety, communication).

### Data collection instruments

A standardized case report form was used to collect all procedural, network, safety, and outcome data prospectively. Post-procedure structured interviews were conducted separately with the remote surgeon, bedside surgeon, and anesthesiologist using validated Likert scales (1‒5) and categorical/open-ended items. The remote surgeon interview included a Raw NASA-TLX (RTLX) workload assessment on five of the six original NASA-TLX dimensions (mental demand, temporal demand, performance, effort, frustration); physical demand was omitted as not informative for a seated, console-based operator in robotic surgery, where physical workload is essentially orthopostural rather than effortful. The pairwise dimension-weighting step was omitted, consistent with RTLX methodology^14^^,^^15^ Responses were recorded on a 21-point visual analogue scale (raw values 0‒20) and rescaled by multiplying by 5 to yield the conventional 0‒100 RTLX score. For mental demand, temporal demand, effort and frustration, higher scores indicate greater perceived workload; for performance, higher scores indicate better self-assessed performance. The interview also included system perception scales, safety and confidence ratings, and clinical acceptability questions.

### Statistical analysis

Given the descriptive nature of this case series (n = 5), results are presented as individual case data and summary statistics (median, range). No inferential statistical tests were performed. Data were tabulated and summarized using Python.

## Results

### Case descriptions

**Fig. 2:**
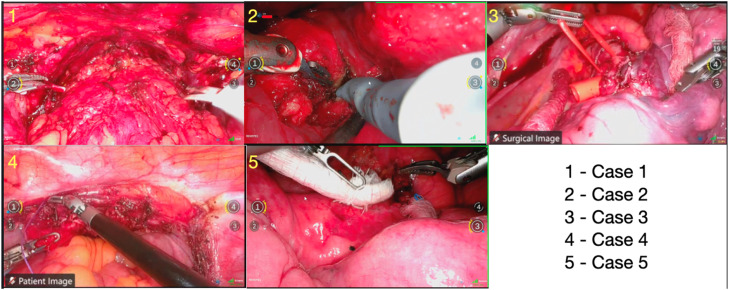
Intraoperative endoscopic snapshots from the five consecutive clinical telesurgeries. Anonymized still frames captured from the surgical console video output during each procedure; panel numbers correspond to case numbers. (1) Radical prostatectomy. (2) Retropharyngeal lipoma resection. (3) Left upper lobe pulmonary segmentectomy. (4) Hysterectomy with bilateral oophorectomy. (5) Resection of thoracic neoplasm.

Five consecutives robotic telesurgeries were performed between February 24, 2026 and March 3, 2026. Table 1 summarizes patient demographics and case characteristics; representative intraoperative snapshots from each procedure are shown in Fig. 2.

**Table 1:** Patient demographics and case characteristics (n = 5).

Case	Age	Sex	BMI	ASA	Specialty	Procedure	Total time (min)	Console time (min)
1	59	M	27.7	2	Urology	Radical prostatectomy	240	196
2	63	M	19.7	2	Head and neck	Retropharyngeal lipoma resection	150	103
3	49	F	33.2	2	Thoracic	Pulmonary segmentectomy (left upper lobe)	210	141
4	63	F	31.0	2	Gynecology	Hysterectomy + bilateral oophorectomy	165	108
5	21	M	21.6	1	Thoracic	Resection of thoracic neoplasm	106	72

#### Case 1

A 59-year-old man (ASA 2) with ISUP grade 2 prostate adenocarcinoma underwent radical prostatectomy. Near the end of the procedure, a local cable disconnection at the patient site interrupted communication between the console and the robotic unit while the network connection itself remained operational; the bedside surgeon assumed control and completed the remaining approximately 10-minutes uneventfully; discharge on postoperative day 1.

#### Case 2

A 63-year-old man (ASA 2) underwent resection of a retropharyngeal lipoma. The procedure was completed via remote console without intraoperative events; the patient developed a Clavien-Dindo grade II complication (edema and pain requiring one additional inpatient day); discharge on postoperative day 2.

#### Case 3

A 49-year-old woman (ASA 2) with primary lung cancer underwent left upper lobe segmentectomy. The procedure was completed via remote console without intraoperative events; discharge on postoperative day 2.

#### Case 4

A 63-year-old woman (ASA 2) with abnormal uterine bleeding due to uterine fibroids underwent hysterectomy with bilateral oophorectomy. A brief power outage at the patient site was buffered by uninterruptible power supply without disrupting the robotic system or network connection; the procedure was completed via remote console; discharge on postoperative day 1.

#### Case 5

A 21-year-old man (ASA 1) underwent thoracic resection of a neoplastic lesion. The procedure was completed via remote console without intraoperative events; discharge on postoperative day 1.

### Network performance

**Fig. 3:**
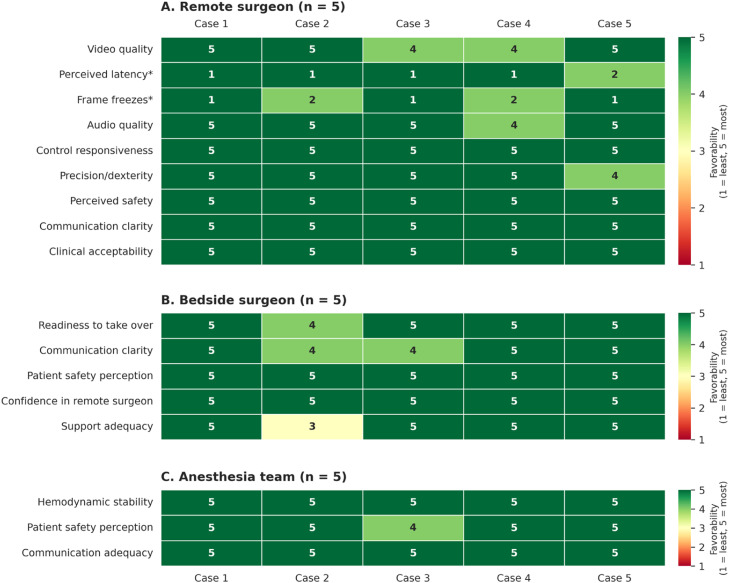
Stakeholder perception scores across the five clinical telesurgery cases. Three vertically stacked heatmaps display Likert scores (1‒5) reported by (A) the remote surgeon, (B) the bedside surgeon, and (C) the anesthesia team for each of the five consecutive cases. Cell color reflects favorability on a unified 1‒5 scale (green = most favorable, red = least favorable); for “Perceived latency” and “Frame freezes” the color is computed on the inverted scale (6 − raw) so that green consistently represents the favorable end across all rows. Cases are ordered chronologically by surgery date.

Console-reported latency was approximately 12 ms in each case ‒ consistent with the wired dry lab phase (median 12.0 ms)^9^ No latency excursions, video freezes or audio dropouts were registered during any procedure. The only command interruption occurred in Case 1, due to the local cable disconnection described above, which prompted conversion to the bedside surgeon. Subjective surgeon perception of latency and freezes was low across all five cases (median 1/5 on a 1‒5 perceived-intensity scale; Fig. 3 panel A).

### Safety events

Across the five cases, no clinical conversion trigger was activated and no intraoperative adverse event was observed. The only technical conversion trigger was the cable issue in Case 1 already detailed above, which led to bedside takeover for the final ∼10-minutes without adverse consequences. A separate, non-telesurgery-related occurrence during Case 4 ‒ a brief power outage at the patient site (HU-USP) ‒ was buffered by the hospital's Uninterruptible Power Supply (UPS), with continuous operation of the robotic system and the network connection maintained throughout.

### Remote surgeon perceptions

Remote surgeon system perceptions across the five cases are summarized graphically in Fig. 3 (panel A). Across all five cases the remote surgeon reported no in-procedure errors and no near-misses, and considered the platform safe for clinical use; the command interruption in Case 1 originated outside the connectivity layer (local cabling) and is described separately (Table 2).

**Table 2:** Remote surgeon workload ‒ Raw NASA-TLX (RTLX, 0‒100 scale).

Domain	Case 1	Case 2	Case 3	Case 4	Case 5	Median
Mental demand	0	15	15	0	15	15
Temporal demand	0	0	15	20	0	0
Performance^a^	100	100	95	100	100	100
Effort	0	0	20	5	5	5
Frustration	0	0	0	0	5	0

a Higher Performance scores indicate better self-assessed performance. Other domains: higher = greater perceived workload. Scores derived from a 21-point visual analogue scale (raw 0‒20) multiplied by 5 to yield the conventional 0‒100 RTLX score.

### Bedside surgeon and anesthesia team perceptions

Bedside surgeon and anesthesia team perceptions across the five cases are summarized graphically in Fig. 3 (panels B and C, respectively). The only full takeover by the bedside surgeon occurred in Case 1, following the protocol-driven conversion described above; in no other case did the bedside surgeon consider proactively assuming the procedure. The model was rated as safe for the patient by all bedside surgeons except in Case 2, where the response was “Depends” ‒ the surgeon attributed the qualification to the receiving hospital lacking adequate material and equipment for head and neck surgery, not to the telesurgery platform itself. The anesthesia team reported no relevant anesthetic events or unplanned anesthetic interventions across all five cases, and unanimously considered the model safe from the anesthetic standpoint. A consolidated visualization of stakeholder perceptions across the three groups is provided in Fig. 3. Most stakeholder perception domains saturated at the maximum Likert value across all five cases, limiting inter-case discrimination and any inference about the responsiveness of the instruments used.

### Postoperative outcomes

Table 3.

**Table 3:** Postoperative outcomes.

Case	Hospital stay (days)	ICU admission	ICU days	Inpatient complications	Clavien-Dindo	30-day complications	30-day Clavien-Dindo	Readmission	Reoperation	Mortality
1	1	No	0	None	0	None	0	No	No	No
2	2	No	0	Edema and pain requiring one additional day of stay	II	None	0	No	No	No
3	2	No	0	None	0	None	0	No	No	No
4	1	No	0	None	0	None	0	No	No	No
5	1	No	0	None	0	None	0	No	No	No

## Discussion

This study reports the first clinical application of robotic telesurgery within Brazil's public health system (SUS), comprising five consecutive procedures across four surgical specialties between PROMIN-FMUSP (remote console) and HU-USP (patient site) via the institutional wired network. Four of five procedures were completed entirely from the remote console; one (radical prostatectomy) required protocol-driven conversion to the bedside surgeon and was completed without intraoperative or postoperative complications attributable to the conversion. One Clavien-Dindo grade II complication occurred (Case 2); no readmission, reoperation or 30-day mortality were recorded, and stakeholder perceptions across remote, bedside and anesthesia teams were favorable, although the saturation of most scores at the maximum value precluded inter-case discrimination.

These findings add clinical validation to the growing body of telesurgery evidence, which has progressed rapidly in recent years. Yang et al. reported a prospective controlled telecholecystectomy trial via 5 G (n = 20 remote, n = 20 local), demonstrating outcomes comparable to conventional robotic surgery^2^ Tai et al. completed a multicenter single-arm phase I trial across China with the EDGE MP1000 system, confirming safety and reliability across multiple sites^7^ Liao et al. extended feasibility to complex hepatic surgery with a prospective remote hepatectomy series^5^ The Toumai platform used in our study has been validated in clinical remote gastrectomy series by Guo et al.^3^ and in the FUTURE-04 prospective trial by Guo et al.,^4^ as well as in a multicentric, multispecialty retrospective study by Sighinolfi et al^16^ More recently, Aldousari et al. reported a series of 11 transcontinental urological telesurgeries across four countries, achieving stable round-trip latencies of 46 to 167 ms over distances up to 7000 km with no clinical or technical adverse events^17^ Bhandari et al. demonstrated the feasibility of tele-robotic bariatric surgery using the indigenous SSI Mantra platform, completing 10 One Anastomosis Gastric Bypass procedures with mean operative times of 59-minutes and no complications^18^ Our series is distinguished from these reports in two respects: 1) It is the first to utilize existing public health system infrastructure rather than dedicated 5 G deployments or private fiber-optic networks; and 2) It captures standardized perceptions from all operating room stakeholders ‒ the remote surgeon, the bedside surgeon, and the anesthesia team ‒ rather than exclusively the remote operator's perspective.

The conversion observed in Case 1 (radical prostatectomy) merits detailed analysis, as it represents the only technical failure in this series and provides important lessons for clinical telesurgery deployment. The cause was a local cable disconnection at the patient site that interrupted communication between the console and the robotic unit; the wide-area network connection itself remained operational throughout. The event occurred near the end of the procedure, and the bedside surgeon assumed full control for the final approximately 10-minutes, completing the operation without complications. This experience underscores two fundamental principles consistently emphasized in the telesurgery literature: the absolute requirement for a qualified bedside surgeon capable of independently completing any ongoing procedure, and the need for a clear, rehearsed conversion protocol.^12^ Beyond these, it highlights an underappreciated dimension of clinical telesurgery readiness: the local apparatus of cabling, connectors and ancillary hardware that bridges the robotic system to the patient introduces additional points of failure independent of the long-haul network. This local apparatus must be subjected to the same redundancy, pre-procedure verification and intraoperative monitoring discipline applied to the connectivity layer itself. Notably, the conversion was orderly and did not compromise patient safety ‒ a finding consistent with the intended behavior of the structured safety framework employed in this study, although a single event cannot establish protocol validity. International guidelines for remote robotic surgery explicitly recommend standby local surgeon capability and standardized handoff protocols as mandatory safety measures, and our experience provides empirical support for these recommendations.^12^

Network performance during the clinical procedures was comparable to the dry lab phase, with the console reporting approximately 12 ms in each case ‒ consistent with the median wired latency observed in Phase 1 (12.0 ms)^9^ No latency excursions, video freezes or audio dropouts were recorded across the five procedures; the single command interruption ‒ the local cable disconnection in Case 1 that necessitated conversion ‒ originated outside the connectivity layer. The decision to restrict the clinical phase to the wired connection ‒ rather than incorporating the private 5 G network tested in Phase 1 ‒ reflected a deliberate risk-minimization strategy during the initial transition to human procedures. The dry lab study had demonstrated that while the private 5 G segment was associated with significantly higher latency (32.4 vs. 12.0 ms) and jitter (30.8 vs. 6.8 ms), task success was preserved^9^ Nevertheless, the priority for the first clinical cases was to employ the network configuration with the most consistent and predictable performance profile. This staged approach mirrors the incremental validation philosophy illustrated by Hara et al.'s 5-year technical and operational consolidation of the hinotori system in Japan, in which clinical deployment followed cumulative validation across communication latency, fail-safe mechanisms and regulatory compliance.8 Future clinical studies incorporating 5 G are planned following consolidation of the wired experience.

The structured safety protocol ‒ comprising predefined conversion triggers and criteria ‒ was fully implemented across all five cases. No latency- or packet-loss-related conversion triggers were activated; the only protocol-driven conversion occurred in Case 1 (cable issue described above), and was executed without adverse consequences. A separate, non-telesurgery-related event during Case 4 ‒ a brief power outage at the patient site ‒ was buffered by the hospital's Uninterruptible Power Supply (UPS), with no interruption of robotic operation or network connectivity, illustrating the relevance of redundant institutional infrastructure for clinical telesurgery. This systematic approach aligns with the expert consensus-based technical guidelines for remote robotic surgery published by Wang et al., which recommend standardized safety frameworks including connectivity monitoring, contingency protocols, and defined roles for local surgical teams.^12^ The recent ethical framework for informed consent in telesurgery proposed by Frenkel et al. further emphasizes the importance of structured disclosure of situational risks, surgeon-specific elements, and shared accountability between remote and local teams ‒ considerations that were addressed in our consent process.^11^

A distinctive contribution of this study is the systematic assessment of perceptions from three independent stakeholder groups involved in clinical telesurgery. While most published series report only the remote surgeon's subjective experience, the bedside surgeon and the anesthesia team play critical and complementary roles in patient safety. In our series, all five remote surgeons rated perceived safety at 5/5, reported no errors or near-miss events, and considered the system suitable for clinical use. Among bedside surgeons, four of five also assigned maximum safety scores, with one rating 4/5 in the head and neck case. This case (Case 2, lipoma of the retropharynx) also elicited a “Depends” response regarding model safety from the bedside surgeon ‒ a concern related to institutional resources rather than the telesurgery system itself. The same surgeon actively assisted during the procedure (instrument placement, suctioning, exposure), which reflects the expected collaborative role of the bedside surgeon in complex cases rather than a failure of remote control. All five anesthesiologists rated hemodynamic stability, patient safety, and communication at 4‒5/5, with no unplanned anesthetic interventions, confirming that from the anesthetic perspective, telesurgical procedures did not differ meaningfully from conventional robotic cases. These convergent perceptions from all stakeholders provide a more comprehensive assessment of clinical telesurgery readiness than surgeon-only evaluations.

A distinct lesson emerged from Case 2 (head and neck) that warrants explicit emphasis. Although the bedside surgeon rated the telesurgery platform itself as safe, the team voiced their concern about possible receiving hospital's institutional readiness for head and neck surgery ‒ specifically the availability of specialty-specific instruments, optics and ancillary equipment. This finding underscores a dimension of clinical telesurgery readiness that extends beyond network performance and platform reliability: the local infrastructure of the receiving site must match the specialty-specific demands of the procedure being performed remotely. For programs aiming to expand surgical access through telesurgery ‒ particularly within heterogeneous public health systems such as SUS, where institutional baseline equipment varies widely between centers ‒ telesurgical scalability is constrained not only by connectivity and credentialing but also by site-level surgical infrastructure. Pre-procedure assessment of the receiving hospital against specialty-specific equipment checklists should be considered a prerequisite alongside network and protocol validation.

The Raw NASA-TLX (RTLX) workload assessment revealed generally low mental demand, effort, and frustration across most cases. Performance scores of 95‒100 on the 0‒100 RTLX scale indicate high self-assessed performance across all cases. Case 3 (pulmonary segmentectomy) showed higher scores for mental demand (15), temporal demand (15), and effort (20), suggesting greater technical difficulty during this procedure despite stable network performance. These case-level variations in workload highlight the importance of procedure-specific and specialty-specific assessments as telesurgery expands across surgical disciplines. Objective neurophysiological correlates of operator cognitive state under network delays ‒ recently demonstrated by Ichihara et al. through brain activity measurements during simulated remote operations^19^ ‒ could complement self-report instruments in future studies.

The multispecialty design of this case series demonstrates feasibility across four specialties under controlled patient selection. The five procedures spanned thoracic surgery (pulmonary nodule resection and segmentectomy), urology (radical prostatectomy), head and neck surgery (retropharyngeal lipoma resection), and gynecology (hysterectomy with bilateral oophorectomy). The gynecologic application has been recently demonstrated in the first European telesurgical hysterectomy reported by Pazzaglia et al., also using the Toumai platform^20^ This diversity aligns with the multicentric experience reported by Sighinolfi et al., who demonstrated Toumai-based telesurgery across multiple specialties,^16^ and extends clinical evidence to specialty-procedure combinations not previously reported in the telesurgery literature. The range of operative times (106 to 240 min) and the variety of anatomical regions confirm that the telesurgery platform maintained stable performance across procedures of differing duration and complexity.

The SUS context adds a critical translational dimension to these findings. Brazil's public health system provides constitutionally guaranteed universal coverage to a population of approximately 203 million^21^ across a continental territory with pronounced regional disparities in access to specialized surgical care ‒ a challenge of inequitable surgical access documented globally by the Lancet Commission on Global Surgery^22^ Demonstrating safe clinical telesurgery between two academic hospitals using the existing institutional wired network ‒ without requiring dedicated 5 G infrastructure or private fiber-optic links ‒ establishes a pragmatic model for initial telesurgery implementation. This approach contrasts with most published series, which rely on dedicated telecommunications infrastructure that may not be scalable to resource-constrained public health settings. As the Chinese experience has demonstrated, systematic institutional protocols and progressive case accumulation are essential for building the operational maturity needed to expand telesurgery safely^23^^,^^24^ An early precedent for sustained clinical telesurgery within a public health system was established by Anvari et al., who developed the world's first telerobotic remote surgical service in rural Ontario nearly two decades ago^25^ The phased approach employed in our program ‒ from dry lab validation (Phase 1)^9^ to supervised initial clinical cases (this study) ‒ provides a replicable template for other public health systems considering telesurgery adoption.

Several limitations must be acknowledged, organized along three dimensions: design and sampling, measurement and instrumentation, and generalization.

Limitations of design and sampling. This is a small, uncontrolled case series (n = 5) without a comparison group, precluding comparative inferences about the relative safety or efficacy of telesurgery versus conventional robotic surgery. Case complexity was deliberately restricted to low-to-medium procedures as a safety measure for initial clinical application; performance in high-complexity surgery remains untested. All procedures were performed on a single robotic platform (Toumai/MicroPort), and findings may not generalize to other telesurgery systems. The robotic platform and surgical instruments were provided by the manufacturer at no cost, and the operating teams simultaneously developed and evaluated the institutional telesurgery program; despite the absence of direct funding, this configuration introduces a potential adoption and reporting bias that should be considered when interpreting the favorable safety and feasibility findings. Eligible patients were not tracked through a formal screening log, so the proportion of patients offered the telesurgical approach who declined is unavailable. Finally, the short-term follow-up (30-days) does not capture potential delayed complications or oncologic outcomes.

Limitations of measurement and instrumentation. Stakeholder perceptions captured represent single-timepoint assessments in a novel clinical context and may be influenced by novelty bias, social desirability, or the Hawthorne effect; in addition, a marked ceiling effect (median 5/5 across nearly all domains) limited inter-case discrimination and underscores the need for instruments with greater dynamic range in future studies. The patient perspective on the telesurgical experience was not collected systematically and should be incorporated as a structured outcome in future studies. Network monitoring was limited to a representative latency reading per procedure and event detection rather than continuous granular logging of jitter and packet loss, precluding detailed correlation between connectivity fluctuations and surgical performance. Conversion criteria were operationalized through the remote surgeon's real-time judgment rather than fixed numerical thresholds, which may limit reproducibility across centers and operators.

Limitations of generalization. All procedures were performed using a wired connection between institutions approximately 5 km apart; generalizability to longer distances, different network architectures, or 5 G configurations is unknown. The single conversion observed (Case 1) demonstrates that technical failures ‒ including those arising from local hardware rather than the long-haul network ‒ do occur, and their frequency and consequences require evaluation in larger series before broader deployment.

Future work should build on this initial clinical experience along five priority lines. First, expansion to larger, prospectively registered series ‒ including multicenter trials at increasing distances within SUS ‒ will enable statistical analysis of safety endpoints and comparison with institutional outcomes for conventional robotic surgery. Second, progressive inclusion of higher-complexity procedures, guided by demonstrated stability at each level, will extend the validated scope of clinical telesurgery. Third, incorporation of the private 5 G network validated in Phase 1 into clinical cases, with continuous real-time logging of latency, jitter and packet loss, will provide more granular evidence on wireless and wired connectivity during human procedures; alternative connectivity options such as low-Earth-orbit satellite links (e.g., Starlink) are also being explored in preclinical thoracic telesurgery models^26^ and merit prospective evaluation. Fourth, the development of a Brazilian regulatory and credentialing framework for clinical telesurgery ‒ building on international consensus guidelines.^12^ and ethical frameworks for informed consent.^11^ ‒ is an essential prerequisite for broader adoption. Finally, health economic analyses comparing the costs and benefits of telesurgery-mediated access expansion with alternative models (e.g., surgeon displacement, patient transfer) will be critical for justifying investment within SUS.

## Conclusion

The first clinical application of robotic telesurgery within the Brazilian public health system proved technically feasible across five consecutive procedures spanning four surgical specialties, with no major adverse events at 30-days. Four of five cases were completed entirely via remote console; one required protocol-driven conversion to local control due to a technical communication failure and was completed without intraoperative or postoperative complications attributable to the conversion. Favorable safety and usability perceptions were reported across all stakeholders. These initial findings ‒ limited by the small sample and absence of a control group ‒ support the continued, progressive evaluation of telesurgery as a tool to address surgical access disparities within SUS.

## Declaration of generative AI in scientific writing

During the preparation of this manuscript, the authors used Anthropic Claude (Opus model family) as a generative artificial intelligence assistant to support text drafting and language refinement. No patient-identifiable data were submitted to the tool, and the AI was not used for data analysis, statistical computation, image generation, or interpretation of clinical results. All AI-assisted content was critically reviewed, verified, and edited by the authors, who take full responsibility for the final content of the manuscript.

## Authors' contributions

Everson L. A. Artifon and Jose Pinhata Otoch served as general coordinators of the project and were responsible for overall study organization and supervision.

Giovanni G. Cerri contributed to the articulation and institutional structuring of the project.

Felipe Kfouri contributed to data collection, manuscript writing, and statistical analysis.

Herbert D. Souza contributed to data collection.

Luiz Paulo Kowalski, William C. Nahas, Paulo Pego-Fernandes, Edmund C. Baracat, Jose Maria Soares Jr., Sergio C. Ribeiro, Marcos Samano, Ricardo Terra, Pedro Nabuco Xavier, Marco A. Kulcsar, Gustavo Ebaid, Eduardo V. Motta, and Alessandro Belon contributed to the organization and coordination of the surgical teams.

## Declaration of competing interest

The robotic telesurgery platform (Toumai/MicroPort) and the surgical instruments used in the procedures were provided by the manufacturer for the purpose of this study. The manufacturer had no role in study design, data collection, analysis, interpretation, or manuscript preparation. No author received personal fees, royalties, equity, or consulting income from MicroPort, and no manufacturer personnel were present in the operating room or in the remote console room during the clinical procedures reported here. The authors declare no other competing interests.
